# Myocardial inflammation in Duchenne Muscular Dystrophy as a precipitating factor for heart failure: a prospective study

**DOI:** 10.1186/1471-2377-10-33

**Published:** 2010-05-21

**Authors:** Sophie Mavrogeni, Antigoni Papavasiliou, Kostas Spargias, Pantelis Constandoulakis, George Papadopoulos, Evangelos Karanasios, Dimitris Georgakopoulos, Genovefa Kolovou, Eftichia Demerouti, Spyridon Polymeros, Loukas Kaklamanis, Anastasios Magoutas, Evangelia Papadopoulou, Vyron Markussis, Dennis V Cokkinos

**Affiliations:** 1 A Cardiology Department, Onassis Cardiac Surgery Center, Athens, Greece; 2 Pediatric Neurology Department, Penteli Children's Hospital, Athens, Greece; 3 Laboratory, Locus Medicus, Athens, Greece; 4 Pediatric Cardiology Department, Agia Sofia Children's Hospital, Athens, Greece; 5 MRI Unit, Bioiatriki Medical Center, Athens, Greece

## Abstract

**Background:**

In patients with Duchenne Muscular Dystrophy (DMD), the absent or diminished dystrophin leads to progressive skeletal muscle and heart failure. We evaluated the role of myocardial inflammation as a precipitating factor in the development of heart failure in DMD.

**Methods:**

20 DMD patients (aged 15-18 yrs) and 20 age-matched healthy volunteers were studied and followed-up for 2 years. Evaluation of myocarditis with cardiovascular magnetic resonance imaging (CMR) was performed using STIR T2-weighted (T2W), T1-weighted (T1W) before and after contrast media and late enhanced images (LGE). Left ventricular volumes and ejection fraction were also calculated. Myocardial biopsy was performed in patients with positive CMR and immunohistologic and polymerase chain reaction (PCR) analysis was employed.

**Results:**

In DMD patients, left ventricular end-diastolic volume (LVEDV) was not different compared to controls. Left ventricular end-systolic volume (LVESV) was higher (45.1 ± 6.6 vs. 37.3 ± 3.8 ml, p < 0.001) and left ventricular ejection fraction (LVEF) was lower (53.9 ± 2.1 vs. 63 ± 2.4%, p < 0.001). T2 heart/skeletal muscle ratio and early T1 ratio values in DMD patients presented no difference compared to controls. LGE areas were identified in six DMD patients. In four of them with CMR evidence of myocarditis, myocardial biopsy was performed. Active myocarditis was identified in one and healing myocarditis in three using immunohistology. All six patients with CMR evidence of myocarditis had a rapid deterioration of left ventricular function during the next year.

**Conclusions:**

DMD patients with myocardial inflammation documented by CMR had a rigorous progression to heart failure.

## Background

Duchenne Muscular Dystrophy (DMD) is a myopathy characterized by a defect in the p21 band of the X chromosome that is responsible for dystrophin, a protein located on the inner surface of the sarcolemma. In affected individuals, the absent or diminished dystrophin leads to progressive skeletal muscle and heart failure [1]. Abnormal dystrophin has also been identified as a potential susceptibility gene for viral infection of the myocardium [2]. Additionally, dystrophin deficiency markedly potentiates the unfavorable course of enterovirus induced cardiomyopathy [3].

Cardiovascular magnetic resonance imaging (CMR) has the unique ability to detect coronary anatomy, function and viability in one examination [4]. The use of a combination of STIR T2-weighted (T2W), T1-weighted (T1W) before and after contrast media and late enhanced images (LGE) can accurately diagnose the presence of myocardial inflammation missed by other imaging techniques [5]. T2W images indicate oedema in myocarditis with a sensitivity of 84% and specificity of 74% [6]. Relative myocardial enhancement and LGE detect myocarditis with a sensitivity of 80% and a specificity of 68% and a sensitivity of 44% and a specificity of 100%, respectively [6]. Recently, late gadolinium enhanced areas were identified in DMD patients and their clinical significance is under evaluation [7,8].

Our aim was to evaluate a population of DMD patients, suspected for myocarditis, using CMR and myocardial biopsy, in order to examine the hypothesis that myocardial inflammation could be a precipitating factor for heart failure in these patients.

## Methods

### Patient population

In this prospective study, twenty consecutive DMD patients (aged 15-18 years) selected from Pediatric Neuromuscular clinics due to a recent onset (last six months) of atypical chest discomfort or fatigue without new ECG changes, were evaluated in our tertiary cardiac center and followed-up for 2 years. All patients were male and presented with proximal muscle weakness before age of 5 years, and increased serum creatine kinase. They were under prophylactic treatment with perindopril since the age of 10 yrs. The diagnosis of DMD was initially based on the characteristic clinical history and neuromuscular findings and was confirmed in all of them by DNA testing. The DMD patients had lost ambulation between 7 and 11 years; they were wheelchair-bound and had moderate to severe scoliosis. All of them had evidence of right ventricular hypertrophy and non-specific ST changes in the ECG and in the Holter monitor examination presented evidence of supraventricular ectopy. Their results were compared with those of 20 age and sex-matched healthy volunteers. In DMD patients with CMR positive for myocardial inflammation, a myocardial biopsy was also considered, as part of standard evaluation of suspected myocarditis.

All subjects (or their parents) gave informed consent. The study was approved by the hospital's ethics committee.

### Methodology

#### A. CMR evaluation of inflammation

Cardiovascular magnetic resonance examination was performed in a 1.5 T Philips Intera system using STIR T2-weighted (T2-W), T1-weighted (T1-W) before and after contrast media injection and late enhanced images (LGE). ECG-triggered, STIR T2-W multislice spin-echo sequence was performed in axial orientation and the signal ratio measured from the region of interest covering the left ventricular myocardium as well as within a skeletal muscle in the same slice. T2-W, an inherent indicator of tissue water content, is increased in inflammation or necrosis due to the development of edema, such as during myocardial infarction or myocarditis.

ECG-triggered T1-W multislice spin-echo images were also obtained in axial orientation with identical parameters before and after an intravenous bolus of 0.1 mmol/kg Gd-DTPA. Measurements after Gd-DTPA were started within 1 minute of injection (early T1) in the same area as in T2-W. The extracellular T1-enhancing contrast agent was used to enhance the detection of pathology on CMR Gd-DTPA. Higher levels of early myocardial enhancement after gadolinium are most likely due to increased cell membrane permeability [6]. This process is an important contributor because inflammation damages cell membrane through both T-cell perforin and B-cell antibody/complement-mediated mechanisms.

Immediately after the second set of T1-W images, 0.1 mmol/kg Gd-DTPA was given again and epicardial late gadolinium-enhanced areas (LGE) were taken 15 min later, using an a 3D-T1-TFE sequence, preconditioned with a 180 degrees inversion pulse (flip angle = 15°, TE = 1.4 msec, TR = 5.5 ms, TI 225 to 275 ms as individually optimized to null myocardial signal, matrix 256 × 192 and slice thickness = 5 mm). Images were analyzed according to previously described protocols [6]. The exact pathophysiology of delayed gadolinium enhancement in myocarditis is still under investigation. Myocardial necrosis in the acute phase appears to play a major role. A combined CMR approach using T2-W, early and late gadolinium enhancement has a sensitivity of 76%, a specificity of 95.5% and a diagnostic accuracy of 85% [6].

#### B. CMR functional study

For each subject, localizing scans were obtained to define the long (2-chamber) axis of the left ventricle. A mid ventricular short axis view was then prescribed, and used to plan a 4-chamber view. The short axis orientation was defined accurately, perpendicular to both the 2- and 4-chamber views. To cover the entire left ventricle, 10 contiguous (gap = 0 mm) short axis slices were acquired in each study. The imaging sequence was a 2D, multi-phase (16 cardiac phases were acquired per cardiac cycle resulting to a temporal resolution of 47 ms for a heart rate of 80 beats/min), steady-state free-precession (SSFP) sequence (TE = 1.5 ms, TR = 3.1 ms, flip angle = 70°, slice thickness = 8 mm, acquired in-plane spatial resolution = 1.8 mm × 2.0 mm) characterized by the application of balanced gradients in all directions.

#### C. Endomyocardial Biopsy (EMB)

After written informed consent, 10 endomyocardial specimens (EMBs) were obtained from the right side of the ventricular septum of each patient with a flexible bioptome (Westmed, Germany) via the femoral vein approach. Four myocardial specimens were used for the histological evaluation according to the Dallas criteria and immunohistochemistry respectively, whereas the remaining 6 were subjected to DNA and RNA extraction of viral genomes.

#### D. Histopathologic analysis

EMBs were stained with hematoxylin-eosin, Masson's trichrome as well as Giemsa stain and examined by light microscopy [8]. For immunohistological identification of cardiac immune cells, tissue sections were treated with an avidin-biotin-immunoperoxidase method according to the manufacturer's protocol (Vectastain Elite ABC Kit, Vector), applying the following monoclonal antibodies: CD3 (T cells, Novocastra Laboratories) and PGM1 (macrophages, Dako).

The detection of >14 infiltrating leukocytes/mm2 in the presence of myocyte damage and or fibrosis in addition to enhanced HLA class II expression in professional antigen-presenting immune cells and endothelium was used for the diagnosis of active myocarditis. Healing myocarditis was considered if the inflammation was less extensive (<14 leukocytes/mm2), whereas healed myocarditis was characterized by multifocal fibrosis or scarring without inflammation (0 to 3 leukocytes/mm2, which is identical to normal myocardium [9].

#### Detection of viral genomes

DNA and RNA were extracted simultaneously from frozen heart muscle tissue probes. Polymerase chain reaction (PCR)/reverse transcriptase (RT)-PCR was performed for the detection of EVs (including Coxsackieviruses and Echoviruses), ADVs, Parvovirus B19 (PVB19), Human Cytomegalovirus, Epstein-Barr-virus, Herpes virus 1-6, Chlamydia pneumoniae and trachomatis. The quality (purity) and quantity of the extracted nucleic acids (DNA, RNA) was evaluated on a Nanodrop 2000c spectrophotometer (ThermoScientific). As a control for the successful extraction of RNAs, as well as for the presence of PCR inhibitors a Reverse Transcription Real Time PCR assay was designed with primer/probe sequences from the G-6PDH (glucose-6-phosphate dehydrogenase) or b2-microglobulin or human acidic ribosomal protein cDNAs (data not shown). For the verification of the DNA extraction a conventional PCR assay was designed with specific primers for the human b-globin gene (IVS-2, data not shown) [10-12].

##### DNA extraction

DNA was extracted with the use of the DNeasy tissue Kit (QIAGEN, BioAnalytica, Athens, Greece) according to the manufacturer's instructions and was stored at -20°C for further processing.

##### RNA extraction

Total RNA was extracted with the use of RNeasy Mini Kit (QIAGEN, BioAnalytica, Athens, Greece) according to the manufacturer's instructions. Total RNA was collected in 40 μl of RNase free water.

#### E. Statistical analysis

Continuous data were expressed as mean ± SD. Statistical analysis was performed using Mann-Whitney U-test. A p-value of < 0.05 was considered for statistical significance.

## Results

In DMD patients, left ventricular end-diastolic volume (LVEDV) was not different compared to controls (96.9 ± 12.9 vs. 101 ± 13.2 ml, p:NS). However, left ventricular end-systolic volume (LVESV) was higher (45.1 ± 6.6 vs. 37.3 ± 3.8 ml, p < 0.001) and left ventricular ejection fraction (LVEF) was lower compared to controls (53.9 ± 2.1 vs. 63 ± 2.4%, p < 0.001). T2 heart/skeletal muscle ratio and early T1 ratio values in DMD patients did not differ compared to controls (1.33 ± 0.23 vs 1.32 ± 0.14 and 3.62 ± 1.47 vs 3.60 ± 0.08, p:NS, respectively).

**Figure 1:**
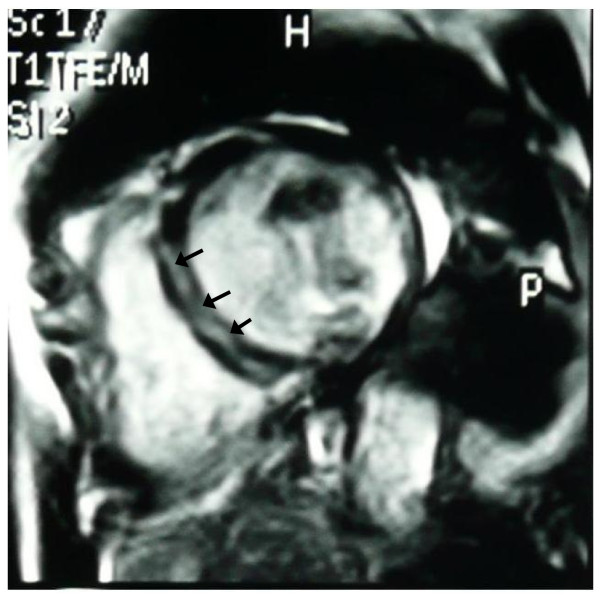
**Intramural late gadolinium enhanced area (LGE) in the intraventricular septum (arrows), indicative of active myocardial inflammation**.

**Figure 2:**
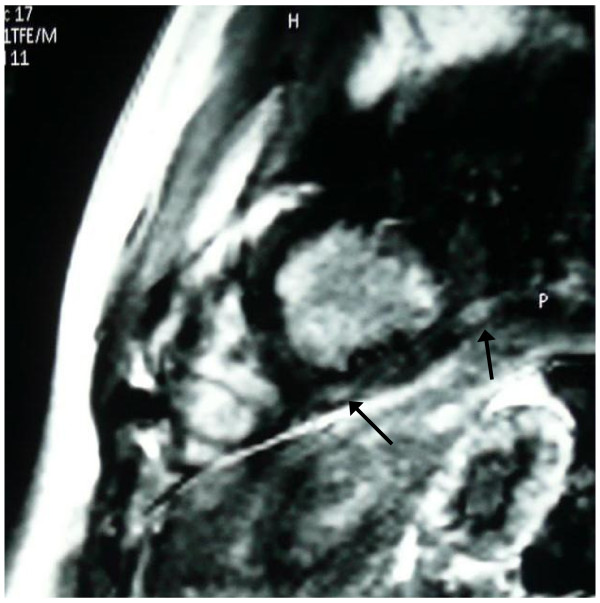
**Epicardial late gadolinium enhanced area (LGE) in the inferior LV wall (arrows), indicative of healing myocarditis**.

However, in one patient, although left ventricular indexes were within normal range, the T2 ratio and early T1 values were increased compared to controls (2.3 and 12 respectively) and a intramural late gadolinium enhanced area (LGE) in the intraventricular septum of the left ventricle was also identified (Figure 1). Epicardial LGE areas in inferolateral wall of left ventricle were also identified in other five DMD patients, although T2 ratio and early T1 were normal (Figure 2).

**Figure 3:**
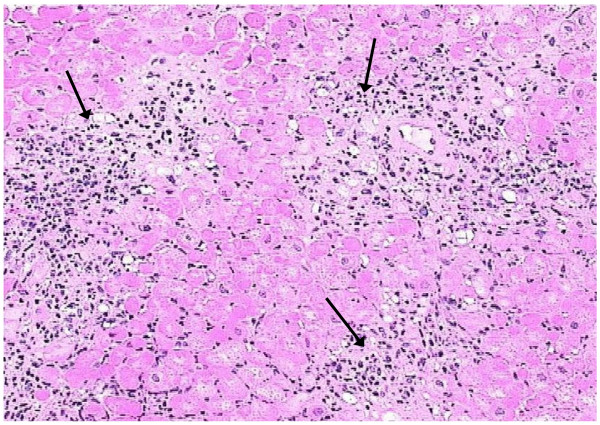
**Pathology image (arrows), indicative of active myocardial inflammation (hematoxylin-eosin, ×200), corresponding to figure 1**.

**Figure 4:**
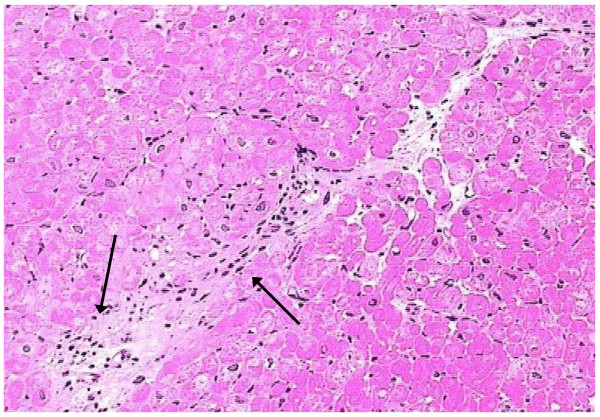
**Pathology image (arrows), indicative of healing myocarditis (hematoxylin-eosin, ×200), corresponding to figure 2**.

In four out of six DMD patients with LGE areas, myocardial biopsy was performed. Evidence of active myocarditis was present in histological analysis in the patient with abnormal T2 ratio, early T1 and positive LGE (detection of >14 infiltrating leukocytes/mm2 in the presence of myocyte damage and or fibrosis) (Figure 3), while evidence of healing myocarditis (<14 leukocytes/mm2), was identified in the other three with positive LGE (Figure 4). LGE areas were not identified in any of the controls.

**Figure 5:**
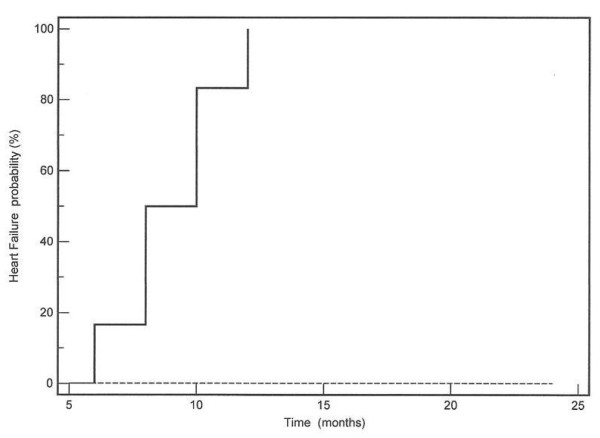
**A Kaplan-Meier graph of the probability of heart failure between LGE+ (continuous line) and LGE- (dotted line) DMD patients**.

Polymerase chain reaction (PCR) revealed Human Cytomegalovirus (CMV) in the patient with active myocarditis, while Parvovirus B19 and Coxsackie B virus were identified in the remaining three patients with postero-lateral LGE areas. During the next year and in a time period between 6-12 months, all 6 DMD patients with CMR evidence of myocarditis (LGE+) developed left ventricular dilatation, rapid deterioration of left ventricular ejection fraction and arrhythmias including bigeminy and/or ventricular tachycardia. In the second year of follow-up, two of them died due to ventricular tachycardia and the other 4 were in end-stage heart failure. It is also important to mention that the DMD patients without evidence of myocarditis (LGE-) remained stable and did not develop any deterioration of heart function during the next 2 years. A Kaplan-Meier graph of the probability of heart failure between LGE+ and LGE- DMD patients is presented in figure 5.

## Discussion

In this study, the evaluation of twenty DMD patients by CMR revealed the presence of myocardial fibrosis in 6/20. DMD patients suspected for myocarditis were oligo-symptomatic and their left ventricular indexes, although within normal range, presented significantly lower than those of age - matched controls. In DMD subjects with positive CMR findings, endomyocardial biopsy assessed the presence of active and healing myocarditis and PCR evaluation documented the presence of CMV, Coxsackie and Parvo-B19 in some of them. During the next 1- 2 years, DMD patients with evidence of myocardial inflammation had a rapid progression to heart failure and death, in contrast to the DMD patients without evidence of myocarditis by CMR, who remained stable.

This is an initial report documenting the presence of myocarditis in a population of oligo-symptomatic DMD patients, using CMR and verified by myocardial biopsy. Our group has already described a case of a DMD patient with sudden clinical deterioration and clinical suspicion of myocarditis (this patient is not included in the present study), initially diagnosed by CMR and then verified by myocardial biopsy [13]. CMR has been previously used as a non-invasive index on tissue structure. In patients with DMD, quantitative maps of relaxation times of peripheral muscles and myocardium were closely related to muscular function and could be used to evaluate the response to treatment [1,14-17].

The cardiac involvement in DMD, which is characterized by cardiac muscle degeneration with fibrous tissue replacement and fatty infiltration, typically occurs late in the course of the disease. It is estimated that about 75% of DMD patients die due to respiratory failure and about 20% of them due to heart failure [18]. These findings could be the result of chronic immobilization [1] or longstanding degenerative process due to the lack or abnormal dystrophin [7,8]. However, in a small percentage of patients, myocardial impairment may progress more quickly than skeletal muscle impairment and lead to sudden heart failure and death in relatively short period of time [18,19].

In an effort to detect early myocardial disease, previous studies used different non-invasive techniques, including 201-Thallium scintigraphy [20] and echocardiography [21]. In a group of DMD patients studied by Corrado et al [22], decreased LV ejection fraction (LVEF), could provide some prognostic value regarding mortality but lacked prognostic ability in a period of five years follow-up. Giglio et al [23] also found that ultrasound tissue characterization can detect preclinical myocardial structural changes in DMD children. Recently, myocardial scintigraphy identified that severe transmural fibrosis and fatty infiltration were common in segments with perfusion defects [24]. Additionally, echocardiography assessed areas of increased echo amplitude in the posterior wall of LV, suspected to be sites of myocardial fibrosis [25]. CMR due to its unique ability to detect function, inflammation and viability in one examination [26] is an excellent diagnostic tool to evaluate the pathophysiology of early myocardial involvement in DMD patients.

Our findings were in agreement with previous studies, which also assessed the presence of epicardial myocardial fibrosis in DMD patients using CMR [6,7] and other non-invasive imaging techniques [24,25]. Furthermore, by performing endo-myocardial biopsy, we documented the presence of histological changes and of viral genomes, which can contribute to myocarditis and precipitate heart failure. Sampling error is a significant limitation to the diagnostic accuracy of the endomyocardial biopsy. Although 4-6 biopsy samples are routinely performed during a diagnostic procedure, a careful post-mortem analysis of proven myocarditis cases demonstrated that > 17 samples were necessary to correctly diagnose myocarditis in >80% of cases [27]. To eliminate the sampling error, we took 10 biopsy samples. Furthermore, it has been well documented that the incidence of myocarditis diagnosed by standard hematoxylin-eosin criteria is underestimated and the Dallas criteria yield diagnostic information in only 10-20% of cases [28]. Growing evidence exists, that the detection of viral genomes by PCR identifies a group of patients with a poorer prognosis, who may benefit from interferon-b therapy [9]. The superimposition of myocarditis on a chronic myocardial disease has been previously commented upon. Kremastinos et al documented the presence of myocarditis in thalassemic patients [29]. Moreover, evidence of myocarditis was also documented in patients with arrythmogenic right ventricle [30]. It is possible that patients with compromised left ventricular structure are more sensitive to viral challenges. However, the detection of these viral genomes in the biopsy supports but does not necessarily prove causation.

Although it is known that the absence or decrease of dystrophin leads to progressive skeletal muscle and heart failure [1], recently has been also documented that abnormal dystrophin can act as a potential susceptibility gene for viral infection of the myocardium [2] and as a factor that markedly increases enterovirus induced cardiomyopathy [3]. The rapid LV deterioration of all our DMD patients with CMR evidence of myocarditis (LGE+) during the next 2 years and the findings of myocardial inflammation and viral genomes in the myocardium further support the hypothesis that myocarditis can act as a precipitating factor for the development of heart failure in DMD patients.

### Limitations of the study

The small number of examined patients and the small number of myocardial biopsies taken from intraventricular septum and not from the gadolinium late enhanced areas are the major limitations of this study. Additionally, our patients were selected due to recent onset of possible cardiac involvement from Pediatric Neuromuscular Hospitals and do not reflect the real incidence of myocarditis in the DMD population.

## Conlusions

In conclusion, in DMD, which is traditionally considered as a degenerative muscular disease, the myocardial inflammation documented by CMR and myocardial biopsy was a precipitating factor for heart failure. DMD patients with myocarditis had rapid LV deterioration in contrary to those without, who remained stable during the 2 years of follow-up. However, further studies are needed to evaluate the true incidence of myocarditis and the importance of these findings in the pathophysiology of development of heart failure in DMD patients.

## Competing interests

The authors declare that they have no competing interests.

## Authors' contributions

SM conceived of the study, and participated in its design and coordination, in the imaging studies and helped to draft the manuscript. AP participated as a pediatric neurologist who followed these patients. KS ED SP performed the cardiac biopsies

PC carried out the PCR studies. GP, EK, DG and GK participated as cardiologists who followed these patients. LK carried out the pathology studies. AM and EP carried out the imaging studies. VM participated in the design of the study and performed the statistical analysis. DVC participated in the design and coordination of the study.

All authors read and approved the final manuscript.

## Pre-publication history

The pre-publication history for this paper can be accessed here:

http://www.biomedcentral.com/1471-2377/10/33/prepub
